# Effect of physical exercise on the subjective wellbeing of middle-aged and older adults: chain mediating role of self-esteem and psychological resilience

**DOI:** 10.3389/fpubh.2026.1804098

**Published:** 2026-05-14

**Authors:** Xi Chen, Menglin Chen, Cheng Miao, Yunhang Lu

**Affiliations:** 1 School of Physical Education and Health Engineering, Taiyuan University of Technology, Taiyuan, China; 2 Department of Public Sports, Soochow College, Soochow University, Suzhou, China; 3 School of Physical Education and Sports Science, Soochow University, Suzhou, China

**Keywords:** chain mediating role, middle-aged and older people, physical exercise, psychological resilience, self-esteem, subjective wellbeing

## Abstract

**Objective:**

The acceleration of population aging has become a major bottleneck constraining improvements in the quality of life of middle-aged and older adults. Enhancing subjective wellbeing is a critical entry point for overcoming this constraint and addressing this developmental challenge. Insufficient physical exercise is associated with subjective wellbeing and physical health among middle-aged and older people. This study aimed to explore the effect of physical exercise on the subjective wellbeing of middle-aged and older adults and examine the chain mediating role of self-esteem and psychological resilience.

**Methods:**

A total of 601 middle-aged and older people were surveyed using physical exercise scale, self-esteem scale, psychological resilience scale, and subjective wellbeing scale. The data were analyzed by reliability analysis, confirmatory factor analysis, common method bias test, correlation analysis, structural equation model, and Bootstrap method to test the mediating effect.

**Results:**

Physical exercise had a significant positive correlation on the subjective wellbeing of middle-aged and older people (*β* = 0.229, *p* < 0.001). Self-esteem (*β* = 0.505, *p* < 0.001, 95%CI = [0.099, 0.238]) and psychological resilience (*β* = 0.271, *p* < 0.001, 95%CI = [0.020, 0.093]) played an independent mediating role in this effect. Self-esteem and psychological resilience (*β* = 0.531, *p* < 0.001, 95%CI = [0.042, 0.118]) played a chain-mediating role in the effect of physical exercise on subjective wellbeing in middle-aged and older people.

**Conclusion:**

Using a chain mediation model, this study explored the relationship between physical exercise and subjective wellbeing among middle-aged and older people, as well as the mediating roles of self-esteem and psychological resilience. The results indicate that physical exercise is significantly and positively associated with subjective wellbeing and that self-esteem and psychological resilience exert a significant chain mediating effect in this relationship. This study not only confirms the important role of physical exercise in enhancing subjective wellbeing but also deepens understanding of the underlying mechanisms, thereby providing a theoretical basis for promoting the physical and mental health of middle-aged and older people.

## 1 Introduction

With the accelerating process of social generational transition, the middle-aged population, which has long served as a backbone of national development, is gradually entering older people. They exhibit a decline in the level of physical function and weakening of self-care ability and experience a decrease in family care and the death of family members, all of which greatly reduce the quality of life and subjective wellbeing of middle-aged and older adults (1). Their physical and mental health status also decreases. As one of the main indicators of an individual’s healthy life, low subjective wellbeing may lead to low mood, decreased self-esteem, and social avoidance in middle-aged and older people, prompting negative emotions, such as loneliness, anxiety, depression, and even psychological disorders, which have a significant negative impact on individual physical and mental health (2). For healthy aging, the health level of middle-aged and older people must be improved, their participation in sports activities must be promoted, and the practical value of physical exercise in promoting physical and mental health development must be maximized (3). Therefore, the impact of physical exercise on middle-aged and older people has become a research hotspot in the academic community.

Physical exercise is a key choice to enhance the wellbeing of the older and enable them to achieve a healthy lifestyle (4). Regular and reasonable participation in physical exercise can effectively improve the stability of the internal environment of the body of middle-aged and older people and is also conducive to their quality of life and subjective wellbeing (5). Physical activity is a beneficial exercise behavior, and subjective wellbeing stems from a variety of factors, especially in the aging population (6). Physical exercise enables middle-aged and older people to cope proactively with life challenges, maintain a positive outlook, improve their quality of life, and enhance both life satisfaction and subjective wellbeing. This, in turn, exerts a significant positive effect on their mental health (7, 8). In related fields, the relationship between physical exercise and subjective wellbeing is mainly explored with a single mediator, while there is relatively little research on the chain mediating mechanism between physical exercise and subjective wellbeing among middle-aged and older adults. Therefore, this study aims to examine the relationship between physical exercise and subjective wellbeing among middle-aged and older adults and analyze the chain mediating mechanism involving self-esteem and psychological resilience. It aims to enrich the theoretical content of the relationship between middle-aged and older adults and subjective wellbeing and provide a reference for improving the mental health of middle-aged and older adults in the context of population aging.

## 2 Literature review and research hypotheses

### 2.1 Effect of physical exercise on subjective wellbeing

Physical exercise is a physical activity that uses physical exercise and exercise load to enhance physical fitness, health, physical and mental development, and physical function (9). Subjective wellbeing is the overall evaluation of an individual’s quality of life and emotional experience according to their own standards (10, 11). Systematic analysis of the relationship between participation in leisure sports activities and positive emotions, negative emotions, and life satisfaction found that participation in sports activities is positively correlated with positive emotions and is conducive to wellbeing. People who regularly participate in physical activity tend to exhibit a positive psychological state, reflect high happiness, and feel the meaning of life (12). Physical exercise can improve the mental health of middle-aged and older adults, thereby promoting their wellbeing (13). The satisfaction that middle-aged and older adults derive from participating in recreational sports activities reflects their overall appraisal of the process, outcomes, and experiences associated with such activities. When they enter a flow state during physical exercise, their satisfaction can increase significantly, which in turn may positively influence subjective wellbeing. Specifically, exercise-related satisfaction may be transformed into enduring positive emotions, thereby enhancing confidence and self-worth; social satisfaction can reduce feelings of loneliness and strengthen the social foundation of wellbeing; and physical satisfaction can improve health beliefs, indirectly increasing life satisfaction and ultimately promoting subjective wellbeing (14). Greater emphasis on health, deeper participation in social capital, improvements in the public sports environment, and an expanded supply of public fitness facilities can effectively encourage individuals to engage actively in physical exercise, thereby facilitating the attainment of subjective wellbeing (15). Aerobic exercises, such as tai chi, can relieve anxiety and depression in middle-aged and older adults and are conducive to their physical and mental health development and subjective wellbeing (16).

### 2.2 Mediating role of self-esteem

As an individual’s overall judgment and attitude towards one’s own value and ability, self-esteem is essentially a subjective perception of self-concept (17). It usually covers two levels: one is the individual’s positive or negative tendencies towards themselves, that is, the degree of self-acceptance or self-loathing; and the other is the overall evaluation of an individual’s own abilities, traits, and achievements, which is often affected by factors such as social environment, cultural background, life experience, and interpersonal communication. From the perspective of social identity theory, the construction and maintenance of a positive social identity can enhance self-esteem and strengthen individuals’ sense of self-worth. In the context of physical exercise, this process may be reflected in active integration into sports groups, a stronger sense of belonging to sports teams, participation in team-based activities, the protection of collective honor, and positive social comparisons with other groups, all of which can effectively enhance self-esteem (18). According to sociometric theory, a high self-esteem has a positive effect on stress relief and negative effects (19). Self-esteem is an individual’s internal perception of self-worth, and its strength depends on the dynamic balance between the actual achievements of the individual in life and his or her own potential ability; the classic self-esteem formula proposes the following: self-esteem = success/ambition (20). Self-esteem is an individual’s perceptual response to the difference between their actual and ideal states. In other words, an individual’s self-esteem depends on their perception of the gap between themselves and their ideal state and the resulting response (21). Physical exercise can help female athletes improve their self-confidence and self-esteem and produce positive psychological effects, which are conducive to their training and performance (22). Physical exercise can also enhance the overall self-esteem of the older adults by improving their loneliness, which is conducive to their participation in physical exercise and social wellbeing (23). The self-esteem and mood state of middle-aged and older adults who participated in winter swimming exercise for a long time were significantly improved, and the mental health effect on this group was also ideal (24). The psychological wellbeing level of older adults with low overall self-esteem was enhanced by participating in physical exercise, indicating that physical exercise plays a moderating role in the relationship between overall self-esteem and psychological wellbeing in the older adults (25). Self-esteem is crucial in relieving stress, helping individuals have positive psychological effects, enhancing self-confidence and enthusiasm to participate in physical exercise, reducing loneliness, and promoting individuals’ subjective wellbeing.

### 2.3 Mediating role of psychological resilience

Mental resilience is an individual’s ability to adapt and maintain or restore mental health when encountering stress or adversity, which is outwardly presented as a tenacious attitude (26). People with high psychological resilience tend to have positive emotional experiences, be able to take the initiative to solve problems, and adopt a positive lifestyle to face difficulties (27). Psychological resilience plays an extremely important role in the lives of middle-aged and older adults and has far-reaching significance for improving their quality of life and maintaining their mental health (28). Physical activity is considered an important factor affecting mental toughness. Regular participation in physical exercise is beneficial to reduce physical and psychological stress, improve the psychological state of individuals, and promote psychological resilience and subjective wellbeing (29). In the face of difficulties in life, middle-aged and older adults with high psychological resilience can use positive emotional experiences to deal with problems; these positive emotional experiences promote their life satisfaction, and their mental health and subjective wellbeing will be better than those of ordinary middle-aged and older adults (30). Psychological resilience has an important impact on the quality of life of middle-aged and older adults: It is essential in alleviating depression and improving sleep quality, overall wellbeing, and mental health (31). Physical exercise can relieve academic pressure, enhance mental toughness and sense of meaning in life, and promote the mental health of college students (32). High psychological resilience is conducive to the psychological state of individuals, helping them participate in physical exercise, relieving depression and anxiety, and enhancing their sense of meaning in life and subjective wellbeing.

### 2.4 Chain mediating role of self-esteem and psychological resilience

Self-esteem, defined as an individual’s overall evaluation of self-worth, is generally regarded as a relatively stable core personality resource, whereas psychological resilience refers to the dynamic process through which individuals adapt to stressful situations. From this perspective, self-esteem can be viewed as an antecedent of psychological resilience. Research has shown that self-esteem not only enhances individuals’ environmental adaptability but also constitutes a key internal component of psychological resilience, thereby serving as an important foundation for resilience (33). Self-esteem significantly and positively predicts psychological resilience, partly through its influence on coping styles. Individuals with higher self-esteem are more likely to interpret stressful situations as challenges rather than threats, which activates more adaptive coping strategies and fosters greater resilience (34). Self-esteem, as a core psychological resource, influences an individua’s cognitive evaluation and coping strategies in stressful situations through the mechanisms of resource conservation theory and self-system theory. Individuals with high self-esteem are more likely to develop positive self-perceptions, adopt adaptive emotion regulation strategies, and employ problem-oriented coping mechanisms, thereby effectively enhancing psychological resilience—a key regulatory capacity. Enhanced psychological resilience further reduces an individual’s social avoidance tendency, ultimately promoting improved social adaptation and behavioral performance (80). High self-esteem alleviates negative emotions caused by difficulties or setbacks, allowing the restoration of psychological balance and enhancing psychological resilience. Especially when encountering stressful events, people with high self-esteem and strong psychological resilience are likely to face problems with a positive attitude and calmly deal with them (35). High self-esteem is positively correlated with good social support; individuals with high self-esteem often adopt positive coping styles when dealing with stressful events, thus promoting their psychological resilience (36). Physical exercise primarily enhances self-esteem by improving body image, self-efficacy, and experiences of achievement, thereby fostering a more positive self-evaluation and stronger sense of self-worth (37). At the same time, exercise strengthens self-efficacy and perceived control, further consolidating the foundations of self-esteem. As self-esteem improves, individuals may become better able to adapt to and recover from setbacks by optimizing cognitive appraisals under stress, strengthening perceived control, and adopting more positive coping strategies. These processes can enhance psychological resilience and support better social adaptation and decision-making (38). According to Conservation of Resources Theory, individuals tend to acquire and preserve key psychological resources, and different resources often accumulate dynamically through a “resource gain spiral.” Within this framework, self-esteem and psychological resilience can be viewed as core psychological resources that mutually reinforce one another: on the one hand, self-esteem helps individuals adopt more effective coping strategies, thereby enhancing psychological resilience; on the other hand, successful experiences of adaptation and recovery can, in turn, strengthen individuals’ self-evaluations. Therefore, a bidirectional relationship may exist between the two constructs (39). It should also be noted that the relationship between self-esteem and psychological resilience is not necessarily a stable one-way pathway. Rather, it may be influenced by factors such as developmental stage, situational stress, and the availability of personal resources. Recent studies have suggested that these psychological structures often exhibit nonlinear and context-dependent dynamic characteristics (40, 41). With the continuous growth of the population aged 45 years and older, health issues among middle-aged adults have received increasing attention. As physical functioning gradually declines, this population becomes more vulnerable to chronic conditions such as hypertension and diabetes, which are often prolonged and complex and can substantially reduce quality of life and subjective wellbeing. Therefore, greater attention should be paid to the physical and mental health of middle-aged adults (42). For middle-aged and older adults, whose wellbeing is shaped by their living environments, strengthening self-esteem and psychological resilience is particularly important. Through physical exercise, their self-confidence and positive self-evaluations can be enhanced, self-esteem can be elevated, quality of life can be optimized, and positive emotions and stability in coping with stress can be improved, thereby facilitating the development of psychological resilience and ultimately promoting subjective wellbeing (43). Individuals with high self-esteem can affirm their self-worth, have the ability to cope and solve problems, quickly adjust their mentality and actions, and continuously learn and improve, eventually developing strong psychological resilience. In physical exercise, individuals can improve their self-esteem by gaining a sense of accomplishment and satisfaction. A solid self-esteem enables individuals to withstand pressure and effectively cope with problems, helping increase their psychological toughness. In this process, individuals continue to grow, ultimately bringing lasting and stable subjective happiness.

**Figure 1:**
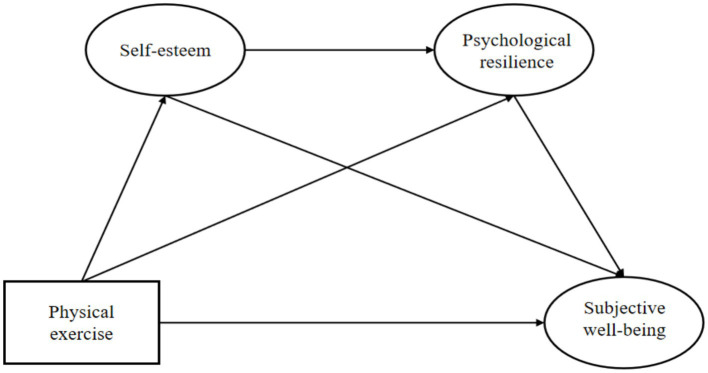
Schematic of the research hypothesis model.

Based on the above analysis, research examining self-esteem and psychological resilience as chain mediators in the relationship between physical exercise and subjective wellbeing remains limited. Clarifying the mechanisms through which physical exercise influences subjective wellbeing among middle-aged and older adults is of considerable importance, as it may provide valuable implications for future research and interventions aimed at improving mental health in this population. Accordingly, this study examines the relationship between physical exercise and subjective wellbeing among middle-aged and older adults, with particular attention to the chain mediating roles of self-esteem and psychological resilience. On this basis, a chain mediation model is constructed, and four hypotheses are proposed to establish the study’s theoretical framework (Figure 1).

*Hypothesis 1*: Physical exercise positively predicts the subjective wellbeing of middle-aged and older adults.

*Hypothesis 2*: Self-esteem acts as a mediator between physical exercise and the subjective wellbeing of middle-aged and older adults.

*Hypothesis 3*: Psychological resilience acts as a mediator between physical exercise and the subjective wellbeing of middle-aged and older adults.

*Hypothesis 4*: Self-esteem and psychological resilience have a chain-like mediating effect between physical exercise and the subjective wellbeing of middle-aged and older adults.

## 3 Methods

### 3.1 Participants

This study employed a convenience sampling. To enhance sample representativeness and ensure coverage across different age groups, participants were middle-aged and older adults from multiple provinces in China. Questionnaires were distributed across 25 provinces, autonomous regions, and municipalities directly under the central government, including Shanxi, Shaanxi, Henan, Hebei, Shandong, Beijing, Heilongjiang, Jilin, Liaoning, Zhejiang, Jiangsu, and Chongqing, among others. Recruitment information was disseminated via WeChat, and the questionnaire QR code was also sent to older adults through the same platform. Participants completed the questionnaire independently using the Wenjuanxing online survey platform. If older adults had difficulty using mobile phones, their children could read the questions aloud, while the participants responded orally and the children assisted with data entry. Data collection was conducted from July 2025 to September 2025. The inclusion criteria were as follows: (1) middle-aged adults aged 45–59 years and older adults aged 60 years or above; (2) clear consciousness, normal communication ability, and the capacity to respond to the questionnaire; and (3) voluntary participation after being informed of the study purpose. The exclusion criteria were as follows: (1) hearing or visual impairment; (2) severe mental illness or other acute exacerbating disorders; and (3) serious diseases involving the heart, kidneys, or other organs. The questionnaire consists of five parts: demographic information, Physical Exercise Scale (PARS-3), Self-esteem Scale (SES), Psychological Resilience Scale (CD-RISC), and Subjective wellbeing Scale (MUNSH). Completion required approximately 20 min. The study followed the principles of informed consent, standardized administration, anonymous completion, and privacy protection to ensure a positive participant experience and effective data collection. A total of 650 questionnaires were distributed and returned. Invalid questionnaires were identified on the basis of contradictory responses, repetitive response patterns, extreme responding, and careless or perfunctory completion (44). After excluding 49 invalid questionnaires, 601 valid questionnaires were retained, yielding an effective response rate of 92.5%. This study was conducted in accordance with the ethical standards set forth in the 1964 Declaration of Helsinki and its subsequent revisions, and was approved by the Ethics Committee of Soochow University (Approval No.: SUDA20250609H19). All participants were informed of the purpose and nature of the study, participated voluntarily, and were assured that their questionnaire responses would remain confidential.

### 3.2 Measuring tools

#### 3.2.1 Demographic information questionnaire

The demographic information questionnaire used in this study included items on gender, age, educational level, marital status, living arrangement, place of residence, income or pension, number of children, current health status, and the types of physical activities regularly performed. No demographic variables were included as covariates in the structural equation model.

#### 3.2.2 Physical exercise scale

The physical activity measurement tool used in this study was originally developed by the Japanese scholar Hashimoto Kimio (45) and later adapted by Liang into the Chinese “Physical Activity Rating Scale” (46). It has been widely used in research involving Chinese populations (47). The scale has three questions that measure the intensity, frequency, and duration of physical activity. Physical activity level was calculated as intensity × duration × frequency. Each question is scored on a 5-point Likert scale; the higher the intensity, frequency, and duration of the exercise, the higher the score. Intensity and frequency levels range from 1 (1 point) to 5 (5 points) and duration from 1 (0 points) to 5 (4 points). The total score on the physical activity scale ranges from 0 to 100. According to the scoring criteria, physical activity levels were classified as low (<=19 points), moderate (20–42 points), and high (> = 43 points).

#### 3.2.3 Self-esteem scale

This study used the Self-esteem Scale developed by Rosenberg in 1965 (21), with item 8 modified (48), one of the most widely used self-esteem measurement tools to assess the self-esteem level of middle-aged and older adults. Previous studies have shown that this scale has also been widely used among middle-aged and older adults in China (49). The scale consists of 10 questions divided into two dimensions: self-affirmation and self-denial. Each question is scored on a 4-point Likert scale (1 = “very disagreed,” 4 = “very conform”). The scale has theoretical scores ranging from 10 points to 40 points, with high scores indicating high levels of self-esteem. The Cronbach *α* of this scale in the present work was 0.899. The KMO value for the Self-esteem Scale was 0.919. Confirmatory factor analysis supported the structure of the questionnaire: χ2/df was 1.540, GFI was 0.983, AGFI was 0.973, SRMR was 0.018, RMSEA was 0.030, NFI was 0.983, TLI was 0.992, and CFI was 0.994, indicating good validity.

#### 3.2.4 Psychological resilience scale

This study used the psychological resilience scale compiled by Connor and Davidson in 2003 (50), translated and revised by domestic scholars Yu and Zhang in 2007 (51, 52). This scale is applicable to the assessment of psychological resilience among Chinese populations (53). It consists of 25 questions covering three dimensions: resilience, self-improvement, and optimism. Each question is scored on a 5-point Likert scale from “never” to “always,” with high scores indicating high levels of mental toughness. The Cronbach *α* of this scale in the present work was 0.965. The KMO value for the Psychological Resilience Scale was 0.981. Confirmatory factor analysis supported the structure of the questionnaire, with χ2/df of 1.123, GFI of 0.962, AGFI of 0.954, SRMR of 0.019, RMSEA of 0.014, NFI of 0.972, TLI of 0.997, and CFI of 0.997, indicating good validity.

#### 3.2.5 Subjective wellbeing scale

This study used the Memorial University of Newfoundland Happiness Scale compiled by Kozma and Stones (54). The scale has been validated among Chinese middle-aged and older adults as a suitable and comprehensive instrument for assessing subjective wellbeing and has also been used in many countries to measure subjective wellbeing and mental health in middle-aged and older populations (55). The scale consists of 24 questions divided into four dimensions: positive affective (PA), negative affective (NA), positive sexual experience (PE), and negative sexual experience (NE). Each question is scored 2 points for answering “yes,” 1 point for “do not know,” and 0 points for “no” using a 3-point Likert scale. The total subjective wellbeing score is calculated as PA-NA + PE-NE, with scores ranging from −24 to +24. For the investigator’s calculation, a constant of 24 is usually added, with scores ranging from 0 to 48. High scores indicate high subjective wellbeing. A score of 36 or above indicates a high level of subjective wellbeing, a score of 12 or below indicates low subjective wellbeing, and a score of 12–36 indicates moderate subjective wellbeing. The Cronbach *α* of this scale in the present work was 0.963. The KMO value for the Subjective wellbeing Scale was 0.967. Confirmatory factor analysis supported the structure of the questionnaire, with χ2/df of 2.172, GFI of 0.930, AGFI of 0.915, SRMR of 0.021, RMSEA of 0.044, NFI of 0.958, TLI of 0.974, and CFI of 0.977, indicating good validity.

### 3.3 Statistical methods

SPSS26.0 software was used for statistical data analysis, and AMOS26.0 software was applied to construct a structural equation model (SEM) and test the mediating effect. First, SPSS26.0 was used for preliminary analysis, and the common method bias test was employed to understand the basic situation of the sample through descriptive analysis. Subsequently, Pearson correlation analysis was conducted to examine the relationships among the variables and the strength of their associations. Finally, AMOS26.0 software was utilized to construct an SEM to test the mediating effect and explore the influence of physical exercise on subjective wellbeing. During model fitting, the fitting indexes of the model (e.g., χ2/df, GFI, AGFI, SRMR, RMSEA, NFI, TLI, and CFI) were checked. The model structure was adjusted according to the fitting results to improve rationality, and the internal relationship between variables was studied through the analysis of the path of structural equations and intermediary testing. The significance of the mediating effect was tested using the bias-corrected Bootstrap program. The influence of independent variables on dependent variables through mediating variables was explained by testing the significance of the mediating effect, thus providing empirical support for the hypothesis of this study.

## 4 Results

### 4.1 Descriptive statistics

The demographic characteristics of the participants in this survey were analyzed descriptively in terms of gender, age, educational level, marital status, living arrangement, place of residence, income or pension, number of children, current health status, and types of physical activities regularly performed. The details are presented in Table 1.

**Table 1:** Descriptive analysis of sample demographics.

Variable	Category	Frequency	Percentage
Gender	Male	288	47.9
	Female	313	52.1
Age	45–59 years old	150	25
	60–69 years old	277	46.1
	70–79 years old	120	20
	80 years old and above	54	9
Educational level	Primary school and below	86	14.3
	Junior high school	242	40.3
	Technical secondary school/High school	165	27.5
	College/Undergraduate	86	14.3
	Above undergraduate	22	3.7
Marital status	Unmarried	148	24.6
	Married	453	75.4
Residential type	Living alone	91	15.1
	Living with a partner	324	53.9
	Living with son or daughter	186	30.9
Residence	Urban	340	56.6
	Rural	261	43.4
Income or pension	Below 3,000 CNY	127	21.1
	3,000–5,000 CNY	310	51.6
	5,000 CNY and above	164	27.3
Number of children	No children	2	0.3
	Only child	195	32.4
	Multiple children	404	67.2
Current health status	Healthy	272	45.3
	Fairly healthy	178	29.6
	Average	103	17.1
	Frailty and frequent illnesses	48	8

As shown in Table 1, the gender distribution was relatively balanced, with females slightly outnumbering males (males: *n* = 288, 47.9%; females: *n* = 313, 52.1%). Regarding age, the largest group was 60–69 years (*n* = 277, 46.1%), followed by 45–59 years (*n* = 150, 25.0%), 70–79 years (*n* = 120, 20.0%), and 80 years or older (*n* = 54, 9.0%). In terms of education, junior high school was the most common level (*n* = 242, 40.3%), followed by technical secondary/high school (*n* = 165, 27.5%), primary school or below (*n* = 86, 14.3%), college/undergraduate (*n* = 86, 14.3%), and postgraduate education or above (*n* = 22, 3.7%). Most participants were married (*n* = 453, 75.4%), whereas 148 (24.6%) were unmarried. Regarding living arrangements, 53.9% lived with a partner (*n* = 324), 30.9% lived with children (*n* = 186), and 15.1% lived alone (*n* = 91). Urban residents (*n* = 340, 56.6%) outnumbered rural residents (*n* = 261, 43.4%). For monthly income/pension, 51.6% reported 3,000–5,000 CNY (*n* = 310), 27.3% reported 5,000 CNY or above (*n* = 164), and 21.1% reported below 3,000 CNY (*n* = 127). Most participants had multiple children (*n* = 404, 67.2%), 32.4% had one child (*n* = 195), and 0.3% had no children (*n* = 2). Regarding self-rated health, 45.3% reported good health (*n* = 272), 29.6% reported fairly good health (*n* = 178), 17.1% reported average health (*n* = 103), and 8.0% reported frailty and frequent illness (*n* = 48).

Table 2 presents the types of physical activities in which participants regularly engaged. Square dancing was the most prevalent activity (*n* = 356, 59.2%), followed by walking/brisk walking (*n* = 334, 55.6%) and badminton (*n* = 292, 48.6%). Long-distance running (*n* = 249, 41.4%) and table tennis (*n* = 243, 40.4%) were also common, whereas participation in other activities was relatively low.

**Table 2:** Frequently participated sports activities.

Categories	Responses		Case percentage	Rank
	Number of cases	Percentage		
Tai Chi	229	7.70%	38.10%	6
Tai Chi sword	207	7.00%	34.40%	7
Square dancing	356	12.00%	59.20%	1
Volleyball	116	3.90%	19.30%	14
Cycling	172	5.80%	28.60%	8
Walking or brisk walking	334	11.20%	55.60%	2
Long-distance running	249	8.40%	41.40%	4
Swimming	157	5.30%	26.10%	10
Rouliqiu (Chinese health ball)	125	4.20%	20.80%	12
Badminton	292	9.80%	48.60%	3
Table Tennis	243	8.20%	40.40%	5
Tennis	160	5.40%	26.60%	9
Shuttlecock kicking	157	5.30%	26.10%	11
Baduanjin (Eight-Section Brocade)	119	4.00%	19.80%	13
Other	61	2.00%	10.10%	15

### 4.2 Common method bias test

Harman’s one-factor test was used to assess the risk of common method bias resulting from the use of a single questionnaire. In the unrotated solution, nine factors with eigenvalues greater than 1 were extracted, accounting cumulatively for 70.297% of the total variance. The first factor explained 37.066% of the variance, which was below the critical threshold of 40%. Because no single factor accounted for most of the total variance, common method bias was not considered a serious threat in this study.

### 4.3 Correlation analysis

According to the Pearson correlation analysis of physical exercise, self-esteem, psychological resilience, and subjective wellbeing, physical exercise was significantly positively correlated with subjective wellbeing (*r* = 0.478, *p* < 0.01), self-esteem (*r* = 0.428, *p* < 0.01), and psychological resilience (*r* = 0.427, *p* < 0.01) (Table 3). Self-esteem was also significantly positively correlated with psychological resilience (*r* = 0.494, *p* < 0.01). Self-esteem and psychological resilience showed a significant positive correlation with subjective wellbeing (self-esteem: *r* = 0.474, *p* < 0.01; psychological resilience: *r* = 0.497, *p* < 0.01). Therefore, assumed H1 was true.

**Table 3:** Correlation analysis of each variable.

Variables	Mean	Standard deviation	Physical exercise	Self-esteem	Psychological resilience	Subjective wellbeing
Physical exercise	34.28	31.139	1			
Self-esteem	2.33	0.804	0.428**	1		
Psychological resilience	3.36	0.873	0.427**	0.494**	1	
Subjective wellbeing	2.14	0.643	0.478**	0.474**	0.497**	1

**p < 0.01.

### 4.4 Structural equation model

#### 4.4.1 Structural model fitting

The calculated model parameters of the established SEM are shown in Table 4. The absolute adaptation index χ2/df of the structural model diagram was 1.298, which is less than 3. GF and AGF were 0.987 and 0.976, respectively, both greater than 0.8. SRMR and RMSEA were 0.017 and 0.022, respectively, both of which are less than 0.08. The NFI of the adaptation index was 0.986, the TLI was 0.955, and the CFI was 0.997, all of which are greater than 0.9. This finding indicates that the fitting effect of the structural model is ideal.

**Table 4:** Structural equation model fit.

Fitting index	Standard of judgment	Fitting result
χ^2^/df	<3	1.298
GFI	>0.8	0.987
AGFI	>0.8	0.976
SRMR	<0.08	0.017
RMSEA	<0.08	0.022
NFI	>0.9	0.986
TLI	>0.9	0.955
CFI	>0.9	0.997

#### 4.4.2 Evaluation of the structural equation model

The path coefficients and significance levels are presented in Table 5. Physical exercise significantly and positively predicted self-esteem (standardized coefficient = 0.505, *p* < 0.001), psychological resilience (standardized coefficient = 0.186, *p* < 0.001), and subjective wellbeing (standardized coefficient = 0.229, *p* < 0.001). Self-esteem also significantly and positively predicted psychological resilience (standardized coefficient = 0.531, *p* < 0.001) and subjective wellbeing (standardized coefficient = 0.318, *p* < 0.001). Moreover, psychological resilience significantly and positively predicted subjective wellbeing (standardized coefficient = 0.271, *p* < 0.001). In terms of the *f*^2^ effect sizes and following Cohen’s standards (*f*^2^ = 0.02 [small], *f*^2^ = 0.15 [medium], *f*^2^ = 0.35 [large]) (56). In this study, the effect size of physical exercise on self-esteem was medium (*f*^2^ = 0.342); the effect size of physical exercise on psychological resilience had a small effect (*f*^2^ = 0.059); the effect size of self-esteem on psychological resilience had a large effect (*f*^2^ = 0.483); the effect size of physical exercise on subjective wellbeing had a small effect (*f*^2^ = 0.098); the effect size of self-esteem on subjective wellbeing was medium (*f*^2^ = 0.189); and the effect size of psychological resilience on subjective wellbeing had a small effect (*f*^2^ = 0.137), further supporting the practical relevance of these relationships.

**Table 5:** Analysis results of structural equation model.

Path	Non-standardized coefficient	Standardization coefficient	S. E.	C. R.	*p*
Physical exercise → self-esteem	0.011	0.505	0.001	10.573	***
Physical exercise → psychological resilience	0.005	0.186	0.001	3.902	***
Physical exercise → subjective wellbeing	0.004	0.229	0.001	5.200	***
Self-esteem → psychological resilience	0.651	0.531	0.076	8.600	***
Self-esteem → subjective wellbeing	0.274	0.318	0.057	4.774	***
Psychological resilience → subjective wellbeing	0.190	0.271	0.040	4.760	***

***p < 0.001.

### 4.5 Mediation effect test

**Figure 2:**
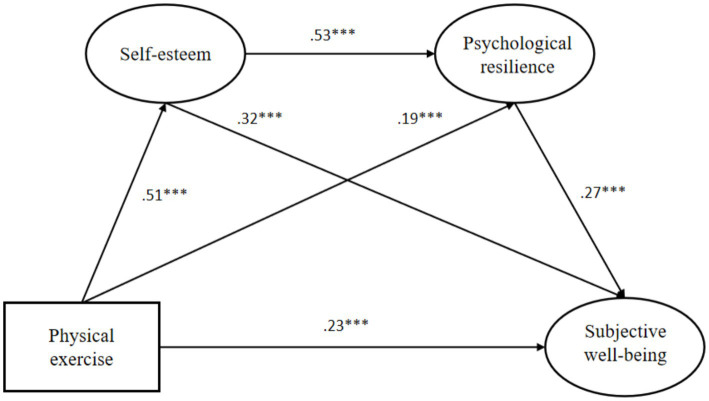
Diagram of the normalization coefficient of the structural model. ***p < 0.001.

The Bootstrap program was used to test the significance of the mediating effect. A mediation model with physical exercise as the independent variable, subjective wellbeing as the dependent variable, and self-esteem and psychological resilience as the mediating variables was established. The mediating effects results of bootstrap analysis are shown in Table 6 and Figure 2. In this work, a Bootstrap sample was selected from the original data (*N* = 601) using repeated random sampling with a confidence interval of 95%. The general thresholds for effect size are as follows: <10% indicates a small effect size, 10–25% indicates a medium effect size, and >25% indicates a large effect size (57, 58).

**Table 6:** Bootstrap test results of the mediating effects of self-esteem and mental resilience between physical exercise and subjective wellbeing.

Effects	Effect size	SE	95% Confidence interval		*p*	Effect proportion (%)
			Lowe limit	Upper limit		
Standardized total indirect effect	0.284	0.035	0.232	0.346	0.000	55.36%
P1: physical exercise → self-esteem → subjective wellbeing	0.161	0.043	0.099	0.238	0.000	31.38%
P2: physical exercise → psychological resilience → subjective wellbeing	0.050	0.021	0.020	0.093	0.003	9.75%
P3: physical exercise → self-esteem → psychological resilience → subjective wellbeing	0.073	0.022	0.042	0.118	0.000	14.23%
Standardized direct effect	0.229	0.048	0.147	0.303	0.000	44.64%
Standardized total effect	0.513	0.034	0.456	0.567	0.000	100.00%

The results showed that on the pathway of “P1: physical exercise → self-esteem → subjective wellbeing,” the confidence interval of 95% of the specific indirect effects was [0.099, 0.238], and no 0 was included, so the mediating effect was significant. The 95% confidence interval for the direct effect of normalization was [0.147, 0.303], excluding 0, so the partial mediating effect was significant. The specific indirect effect value was 0.161, the total effect value was 0.513, and the mediating effect ratio was 0.161/0.513 = 0.3138. This finding indicates that, on the P1 pathway, part of the effect of physical exercise on subjective wellbeing is transmitted through the mediating role of self-esteem, which accounts for 31.38% of the total effect (large effect size). So the mediating effect of self-esteem on physical exercise and subjective wellbeing was significant, and H2 was assumed to be true. In the pathway of “P2: physical exercise → psychological resilience → subjective wellbeing,” the 95% confidence interval for the specific indirect effect was [0.020, 0.093], and no 0 was included, so the mediating effect was significant. The direct effect of standardization was significant, so the partial mediating effect was also significant. The specific indirect effect value was 0.050, the total effect value was 0.513, and the mediating effect ratio was 0.050/0.513 = 0.0975. Therefore, when physical exercise had an impact on subjective wellbeing on the P2 pathway, part of this effect is transmitted through the mediating role of psychological resilience, which accounts for 9.75% of the total effect (small effect size). So the mediating effect of mental toughness on physical exercise and subjective wellbeing was significant, and H3 was assumed to be true. In the pathway of “P3: physical exercise → self-esteem → psychological resilience → subjective wellbeing,” the confidence interval of 95% of the specific indirect effects was [0.042, 0.118], no 0 was included, so the mediating effect was significant. The direct effect of standardization was significant, the partial mediating effect was also significant. The specific indirect effect value was 0.073, the total effect value was 0.513, and the mediating effect ratio was 0.073/0.513 = 0.1423. Hence, when physical exercise had an impact on subjective wellbeing on the P3 pathway, part of this effect operates through the influence of self-esteem on psychological resilience, accounting for 14.23% of the total effect (medium effect size). So the chain mediating effect of self-esteem and psychological resilience in physical exercise and subjective wellbeing was significant, and H4 was assumed to be true.

## 5 Discussion

This study investigated the relationship between physical exercise and subjective wellbeing among middle-aged and older adults and examined the chain mediating roles of self-esteem and psychological resilience through a mediation model. The results showed significant positive correlations among physical exercise, self-esteem, psychological resilience, and subjective wellbeing. Further analyses revealed that self-esteem and psychological resilience not only played independent mediating roles but also exerted a chain mediating effect in the relationship between physical exercise and subjective wellbeing. Specifically, physical exercise was positively associated with self-esteem, which in turn promoted psychological resilience, and psychological resilience contributed to enhanced subjective wellbeing, thereby jointly influencing the physical and mental health of middle-aged and older adults.

### 5.1 Effect of physical exercise on the subjective wellbeing of middle-aged and older adults

The above results show that physical exercise significantly positively predicts the subjective wellbeing of middle-aged and older adults. The higher the level of physical exercise, the higher the subjective wellbeing of middle-aged and older adults, consistent with the research results of domestic (14) and foreign scholars (59). However, study have reported that the relationship between physical exercise and subjective wellbeing is fully mediated by exercise identity. In other words, when exercise identity is controlled for, the relationship between physical exercise and subjective wellbeing disappears (60). This differs from the findings of the present study. Nevertheless, most previous findings are consistent with those reported here. As an appropriate form of exercise for middle-aged and older adults, Tai Chi helps enhance cognitive function and quality of life and serves as an important factor in supporting wellbeing by promoting higher levels of subjective wellbeing (61). As a spontaneous sport of the masses, square ballroom dance is less affected by the venue and is suitable for the exercise needs of middle-aged and older adults. It not only contributes to the physical and mental health of older adults and is closely related to their quality of life, but also facilitates interpersonal communication and helps enhance their subjective wellbeing (62). The level of physical exercise is associated with multiple dimensions in middle-aged and older adults, including emotional state, executive function and memory performance, brain-derived neurotrophic factor levels, cerebral blood flow perfusion, neuroplasticity indicators, as well as cardiopulmonary function and cardiovascular health. Overall, it is linked to higher levels of subjective wellbeing. From a mechanistic perspective, coordinated changes in these factors may constitute an important physiological and psychological basis for the relationship between physical exercise and subjective wellbeing. Further analysis suggests that individuals with higher levels of physical exercise often demonstrate better cognitive function, self-efficacy, and social participation, while exhibiting fewer negative psychological characteristics, such as rumination, anxiety related to cognitive decline, and loneliness. In summary, these variables appear to form a mutually coupled pattern of association that collectively benefit the physical and mental health and subjective wellbeing of middle-aged and older adults (63, 64). Therefore, the positive relationship between physical exercise and subjective wellbeing among middle-aged and older adults is further supported.

### 5.2 Independent mediating role of self-esteem between physical exercise and subjective wellbeing of middle-aged and older adults

The SEM results indicate that, in addition to a significant direct positive association, physical exercise is also indirectly and positively associated with subjective wellbeing among middle-aged and older adults through self-esteem. In other words, self-esteem mediates the relationship between physical exercise and subjective wellbeing in this population. However, study have suggested that although self-esteem is an important mediator in the relationship between physical activity and subjective wellbeing, its indirect effect is relatively weaker than those of perceived health and social support, indicating that self-esteem may not be the primary explanatory mechanism in this pathway (65). Nevertheless, most previous findings are consistent with those of the present study. Self-esteem is widely studied in sports psychology, and improvements in self-esteem can effectively predict physical and mental health. Physical exercise is an important means of enhancing self-esteem. The higher an individual’s physical fitness and self-esteem, the greater their overall confidence and self-worth, which may lead to higher subjective wellbeing (66). Self-esteem is the core internal factor affecting subjective wellbeing; people with high self-esteem have a stable recognition of their self-worth and are likely to maintain a positive attitude in the face of setbacks, preventing the consumption of negative emotions on happiness (67). Greater engagement in physical exercise is associated with higher levels of self-esteem, a stronger sense of meaning in life, and greater subjective wellbeing among middle-aged and older adults (68), which is consistent with the results of this study. Physical exercise is directly and positively associated with subjective wellbeing among middle-aged and older adults and may also be indirectly related to their subjective wellbeing through enhanced self-esteem.

### 5.3 Independent mediating role of psychological resilience between physical exercise and subjective wellbeing of middle-aged and older adults

This study demonstrates that physical exercise is significantly and positively associated with subjective wellbeing among middle-aged and older adults and can also exert an indirect positive association through psychological resilience. In other words, psychological resilience plays a mediating role between physical exercise and subjective wellbeing in this population. However, study have shown that the effects of physical activity on subjective wellbeing mainly operate through social support and self-esteem. Among these, social support constitutes the strongest mediating pathway, whereas self-esteem plays a secondary and relatively weaker explanatory role. Although resilience was included in those models, it did not emerge as a key mediator in the relationship between physical activity and subjective wellbeing. These findings suggest that the mechanism linking physical activity and subjective wellbeing may depend more heavily on social and self-evaluative pathways, with resilience not serving as the core mediating variable (69). Nevertheless, many other previous studies are consistent with the results of the present study. Participation in outdoor sports activities is positively associated with perceived health and psychological resilience. Notably, higher levels of participation in outdoor sports, together with better perceived health and greater psychological resilience, may effectively enhance subjective wellbeing among older adults (70). Studies have also shown that individuals who engage more frequently in physical exercise tend to exhibit a more favorable combination of psychological adaptability and subjective wellbeing, suggesting a synergistic pattern of improvement. Among older adults living alone, those who regularly participate in physical exercise generally report higher levels of psychological resilience. Their psychological state tends to be more resilient and autonomous, which is generally associated with higher subjective wellbeing (71). Physical exercise is significantly related to psychological resilience among middle-aged and older adults. Greater participation in physical exercise is associated with stronger psychological adjustment and recovery, a greater sense of life meaning, a more positive mindset, and, indirectly, higher subjective wellbeing (72). In conclusion, physical exercise has a direct positive association with subjective wellbeing among middle-aged and older adults and may also be indirectly related to subjective wellbeing through psychological resilience.

### 5.4 Chain mediating role of self-esteem and psychological resilience between physical exercise and the subjective wellbeing of middle-aged and older adults

The results of this study show that self-esteem and psychological resilience have a chain mediating effect on the relationship between physical exercise and subjective wellbeing in middle-aged and older adults. This process essentially reflects a gradual and dynamic chain of associations. Specifically, higher levels of physical exercise are generally accompanied by higher self-esteem, greater social participation, and stronger self-confidence. At the same time, self-confidence shows a progressive association with psychological resilience, thereby forming an overall virtuous cycle. The interconnection among these variables ultimately contributes to the subjective wellbeing of middle-aged and older adults. However, study have suggested that psychological resilience may precede self-esteem and significantly and positively predict it. Higher psychological resilience may effectively enhance an individual’s level of self-esteem. In such a chain mediation model, psychological resilience serves as the antecedent variable and influences depression by improving self-esteem, thereby forming a continuous mediating pathway. These findings indicate that psychological resilience is an important protective factor for self-esteem and that the two are consistently and positively associated (73). Nevertheless, most previous findings are consistent with those of the present study. The greater the level of protective factors an individual possesses, the stronger their psychological adaptability. As a result, they may experience less negative impact when facing difficulties such as loneliness and may approach life with a more positive attitude, which is related to higher subjective wellbeing (74). Regular physical exercise is positively associated with higher levels of exercise satisfaction and value identification; middle-aged and older adults who frequently engage in physical exercise tend to report a stronger sense of meaning in life, higher self-esteem, and certain advantages in psychological resilience (75). Therefore, the chain mediating role of self-esteem and psychological resilience between physical exercise and subjective wellbeing in middle-aged and older adults has been further strengthened. This model has the limitation of omitting potential moderating variables. In the present analysis, important moderators such as social support and stress were not included, which may reduce the explanatory power and stability of the model.

### 5.5 Research significance

At the physiological level, regular physical exercise is a central means of maintaining the physical health of middle-aged and older adults and is significantly negatively associated with the risk of chronic disease. It may predict better physical functioning, cardiopulmonary function, muscle strength, and metabolic status, thereby positively contributing to the overall health of this population (76). Participation in ball sports is positively associated with improved hand-eye coordination, reaction speed, and balance, which may help delay cognitive decline (77). The favorable physical condition resulting from regular exercise is also significantly and positively associated with lower health burdens, greater mobility, and higher self-esteem among older adults. It further demonstrates a positive covariation with daily participation, social activity levels, and subjective wellbeing, thereby providing a cross-sectional evidence for understanding the physiological factors related to subjective wellbeing.

At the psychological level, physical exercise is an important means through which middle-aged and older adults can build psychological capital and optimize emotional experiences. Participating in physical exercise not only directly generates positive emotions but may also be indirectly associated with subjective wellbeing through various psychological mechanisms. On the one hand, the more actively individuals participate in physical exercise, the greater their confidence in physical activity, the more positive emotions they experience, and the more frequent their social interactions become. Through these experiences, individuals may gain a sense of achievement and self-identity, which helps improve self-evaluation and self-esteem (78). On the other hand, physical exercise may positively predict an individual’s psychological adaptability, alleviate feelings of helplessness and anxiety, and be significantly positively associated with improvements in the ability of middle-aged and older adults to cope with stress and setbacks, enabling them to maintain a more positive and stable attitude when facing life challenges (79). Furthermore, physical exercise shows a significant positive covariation with social support and self-efficacy. Group sports settings help strengthen interpersonal connections and a sense of belonging, show a significant negative association with loneliness, and maintain a stable positive association with mental health and subjective wellbeing.

In practical terms, physical exercise programs tailored to middle-aged and older adults should be actively promoted, and this population should be encouraged to adopt a lifestyle of regular exercise. Governments and communities should promote group-based physical activities such as square dancing and brisk walking, recommending 3–5 sessions per week at a moderate intensity. They should also establish community fitness stations and activity centers and implement group exercise programs to enhance participation, reduce loneliness, and improve mental health and wellbeing. Through sustained engagement in physical exercise, self-esteem and psychological resilience among middle-aged and older adults can be systematically enhanced, subjective wellbeing can be promoted at multiple levels, and the coordinated development of physical health and psychological wellbeing can be achieved.

## 6 Research limitations and future research prospects

This study explores the influence mechanism of physical exercise on subjective wellbeing in middle-aged and older adults, especially the chain mediating role of self-esteem and psychological resilience in this process. This complex relationship can provide a theoretical basis and practical reference for future research. However, this study still has certain limitations. It needs to be improved in the future.

### 6.1 Research limitations

(1) Because this study employed a cross-sectional design, causal relationships among the variables cannot be established with certainty, and other unexamined factors may also be involved. (2) The questionnaire data in this study were collected using a self-report method, which is susceptible to social desirability bias and self-selection bias. Participants may overestimate indicators such as self-esteem and psychological resilience, thereby interfering with the relationships between variables and affecting the rigor of the results. (3) This study did not adequately consider potential moderating variables such as social support, stress levels, and coping styles, which may influence the relationship between self-esteem and psychological resilience. Furthermore, the study did not control for demographic variables such as gender, age, education level, and housing status as covariates, which may potentially interfere with the results regarding subjective wellbeing in middle-aged and older adults. (4) The sample was recruited via Wechat, which may have introduced sampling bias. This approach may create systematic differences in internet use, educational level, and age structure among participants, thereby limiting the representativeness and generalizability of the findings. (5) This study was conducted within the Chinese cultural context. Self-esteem and psychological resilience are culturally specific, and their structure and mechanisms of action may differ across cultures, thus limiting the cross-cultural applicability of the results.

### 6.2 Future research prospects

Future research may employ longitudinal designs or experimental interventions to verify the dynamic relationship between physical exercise, self-esteem, psychological resilience, and subjective wellbeing, thereby strengthening causal inferences. Simultaneously, multi-source data from external assessments could be incorporated, combining objective indicators with subjective reports to reduce bias. A multi-level moderation framework can be constructed based on a chain mediation model, controlling for demographic covariates to improve the accuracy of the results. Furthermore, multi-channel sampling combining online and offline methods will enhance sample representativeness, and cross-regional cultural comparisons will be conducted to strengthen the generalizability of the conclusions.

## 7 Conclusion

This study explored the relationship between physical exercise and subjective wellbeing among middle-aged and older adults through a chain mediation model and further examined the potential mediating roles of self-esteem and psychological resilience in this relationship. The results indicate that physical exercise is positively associated with subjective wellbeing among middle-aged and older adults. Moreover, self-esteem and psychological resilience exert a significant chain mediating effect in the relationship between physical exercise and subjective wellbeing. These findings not only affirm the central role of physical exercise in enhancing subjective wellbeing but also contribute to the promotion of physical and mental health among middle-aged and older adults. In addition, the study provides a new perspective for future research, enriches the existing literature, deepens understanding of the internal mechanism linking physical exercise and subjective wellbeing, and offers a theoretical basis for applying physical exercise in the field of physical and mental health among middle-aged and older adults.

## Data Availability

The raw data supporting the conclusions of this article will be made available by the authors, without undue reservation.
